# Evaluating context effects on PHQ-8 somatic item scores among people with a chronic medical condition: a scleroderma patient-centred intervention network randomised experiment

**DOI:** 10.1017/S204579602610047X

**Published:** 2026-02-23

**Authors:** Sophie Hu, Marie-Eve Carrier, Meira Golberg, Marie -Claude Geoffroy, Linda Kwakkenbos, Susan J. Bartlett, Catherine Fortuné, Amy Gietzen, Karen Gottesman, Amanda Lawrie-Jones, Vanessa L. Malcarne, Michelle Richard, Maureen Sauvé, Luc Mouthon, Andrea Benedetti, Brett D. Thombs

**Affiliations:** 1 Lady Davis Institute for Medical Research, Jewish General Hospital, Montreal, QC, Canada; 2 Department of Epidemiology, Biostatistics, and Occupational Health, McGill University, Montreal, QC, Canada; 3 Douglas Mental Health University Institute, Montreal, QC, Canada; 4 Department of Psychiatry, McGill University, Montreal, QC, Canada; 5 Department of Clinical Psychology, Behavioural Science Institute, Radboud University, Nijmegen, the Netherlands; 6 Radboudumc Center for Mindfulness, Department of Psychiatry, Radboud University Medical Center, Nijmegen, the Netherlands; 7 Department of Medicine, McGill University, Montreal, QC, Canada; 8 Research Institute of the McGill University Health Centre, Montreal, QC, Canada; 9 Ottawa Scleroderma Support Group, Ottawa, ON, Canada; 10 Steffens Scleroderma Foundation, Albany, New York, NY, USA; 11 National Scleroderma Foundation, Los Angeles, CA, USA; 12 Scleroderma Australia, Melbourne, VIC, Australia; 13 Scleroderma Victoria, Melbourne, VIC, Australia; 14 Department of Psychology, San Diego State University, San Diego, CA, USA; 15 San Diego Joint Doctoral Program in Clinical Psychology, San Diego State University/University of California, San Diego, CA, USA; 16 Scleroderma Atlantic, Halifax, NS, Canada; 17 Scleroderma Society of Ontario, Hamilton, ON, Canada; 18 Scleroderma Canada, Hamilton, ON, Canada; 19 Service de Médecine Interne, Centre de Référence Maladies Autoimmunes et Autoinflammatoires Systémiques Rares d’Ile de France, de l’Est et de l’Ouest, Hôpital Cochin, Paris, France; 20 Assistance Publique Hôpitaux de Paris-Centre, Hôpital Cochin, Université Paris Cité, Paris, France; 21 Respiratory Epidemiology and Clinical Research Unit, McGill University Health Centre, Montreal, QC, Canada

**Keywords:** assessment, depression, randomised controlled trial, somatic symptoms, systemic sclerosis

## Abstract

**Aims:**

Assessing depression symptoms in people with a chronic illness is challenging due to possible bias from overlapping somatic symptoms associated with both depression and chronic illnesses. Previous studies, however, have found that people with a chronic illness do not report more somatic symptoms on depression measures than people without a chronic illness with similar levels of mood and cognitive symptoms. The reason for this surprising finding is unknown. Our primary objective was to evaluate differences in mean sum scores of Patient Health Questionnaire-8 (PHQ-8) somatic symptom items (sleep disturbances, fatigue, appetite changes) in people with a chronic illness when the items were administered outside the context of a depression questionnaire versus as part of the PHQ-8. Secondary objectives were to evaluate individual somatic item scores. We hypothesised that people who completed somatic items outside of a depression assessment would have significantly higher scores than those who completed items as part of a depression assessment.

**Methods:**

We conducted a randomised controlled experiment within the Scleroderma Patient-centred Intervention Network (SPIN) Cohort, a multinational cohort of people with systemic sclerosis. SPIN Cohort participants were randomly allocated to complete the PHQ-8 with somatic items (sleep disturbances, fatigue, appetite changes) presented separately from psychological items and without any indication that they were part of a depression questionnaire (Reordered Items arm) or in standard format (Standard PHQ-8 arm). Participants were automatically randomised when they logged into the SPIN Cohort platform to complete routine research assessments. The primary outcome was the mean sum score of PHQ-8 somatic items. Secondary outcomes were the mean scores of individual somatic items. Differences were assessed using between-groups *t*-tests.

**Results:**

In total, 851 participants were included (*N* = 428 in Reordered Items arm, *N* = 423 in Standard PHQ-8 arm). Mean (SD) PHQ-8 score was 6.0 (5.3) for all participants. We found no statistically significant differences in PHQ-8 somatic item sum scores (0.05 points; 95% confidence interval [CI]: −0.29 to 0.38) or in mean scores for item 3 (sleep disturbances; 0.04 points; 95% CI: −0.09 to 0.19), item 4 (fatigue; 0.03 points; 95% CI: −0.11 to 0.16) and item 5 (appetite changes; −0.03 points; 95% CI: −0.15 to 0.10).

**Conclusions:**

We did not find evidence that responses to PHQ-8 somatic items were influenced by whether participants were aware they were responding to items about depression. This finding supports the validity of self-reported questionnaires for depression symptom assessment in people with chronic medical conditions.

## Introduction

Major depression symptoms, as described in the Diagnostic and Statistical Manual of Mental Disorders, Fifth Edition (DSM-5), include cognitive and mood-related psychological symptoms as well as somatic symptoms, including fatigue, poor appetite or overeating and insomnia or hypersomnia (American Psychiatric Association, 2013). Most self-report depression symptom questionnaires include items that reflect both psychological and somatic symptoms (Sakakibara *et al.*, 2009; Wakefield *et al.*, 2015). Scores obtained using such questionnaires, however, may be artefactually inflated in people with a chronic illness due to the overlap of somatic symptoms related to depression and those stemming from their physical illness (von Ammon Cavanaugh, 1995; Koenig *et al.*, 1997; Sørensenf *et al.*, 2005).

The 9-item Patient Health Questionnaire-9 (PHQ-9) (Kroenke *et al.*, 2001) and its 8-item version (PHQ-8) (Wu *et al.*, 2020) are commonly used for identifying people who may have depression (Moriarty *et al.*, 2015; Levis *et al.*, 2019) and as continuous measures to assess depression symptom severity (Kroenke *et al.*, 2001). The 9 items of the PHQ-9 align with the 9 DSM-5 symptom criteria for a major depressive episode (American Psychiatric Association, 2013), and the PHQ-8 includes all PHQ-9 items except an item on thoughts of suicide or self-harm (Wu *et al.*, 2020). In contrast to what might be expected, existing research suggests that having a medical illness and, among people with a medical illness, condition severity are not associated with higher PHQ-9 somatic symptom item scores (Leavens *et al.*, 2012; Jones *et al.*, 2015; Cook *et al.*, 2017; Hu and Ward, 2017; Marrie *et al.*, 2023). Differential item functioning (DIF) analyses assess the degree to which questionnaire items are likely to measure the intended construct and not be influenced by other external factors (Walker, 2011). If DIF based on having a chronic illness were present, people with an illness would score higher on PHQ-9 somatic symptom items than people without an illness with similar psychological symptom item scores, presumably due to overlap with their medical symptoms.

We identified 5 studies on DIF in the PHQ-9 (Leavens *et al.*, 2012; Jones *et al.*, 2015; Cook *et al.*, 2017; Hu and Ward, 2017; Marrie *et al.*, 2023), including 4 that compared people with and without a medical illness (Leavens *et al.*, 2012; Cook *et al.*, 2017; Hu and Ward, 2017; Marrie *et al.*, 2023) and one that examined whether having more cancer-related somatic symptoms was associated with PHQ-9 somatic item responses (Jones *et al.*, 2015). None of the studies found detectable DIF on any somatic items that meaningfully influenced total measure scores. One of the studies (Leavens *et al.*, 2012) was conducted among people with systemic sclerosis (SSc; also known as scleroderma), a complex, rare, chronic, autoimmune disease characterised by microvascular damage and fibrosis of the skin and other organs, including the lungs, gastrointestinal tract, kidneys and heart (Allanore *et al.*, 2015; Denton and Khanna, 2017; Volkmann *et al.*, 2023). Disease onset typically peaks around age 50, and over 80% of people with SSc are women (Allanore *et al.*, 2015). Common symptoms include skin thickening, Raynaud’s phenomenon, difficulty breathing, limitations in hand mobility, and 3 symptoms measured by PHQ-9 items: fatigue, sleep problems and gastrointestinal issues that can lead to changes in appetite (Allanore *et al.*, 2015; Denton and Khanna, 2017; Volkmann *et al.*, 2023).

It is possible that people with somatic symptoms from medical conditions do not score higher on somatic symptom items than people without medical conditions due to item-order or context effects. Item-order or context effects occur in questionnaires when the order in which items are presented provides a context that affects how people interpret and respond to items (Bowling and Windsor, 2008; Lietz, 2010; Thau *et al.*, 2021; Lee *et al.*, 2023). If people with a chronic illness with substantial somatic symptom burden are aware that they are answering questions about depression, they might not report overlapping somatic symptoms because they attribute them entirely to their physical illness and not to depression, which is more often associated with cognitive and mood-related symptoms. If this were the case, these people would theoretically score higher on items measuring somatic symptoms of depression if these items were removed from the context of a depression questionnaire.

We did not identify any studies that have examined whether similar reporting of somatic symptoms on depression symptom questionnaires between people with and without medical conditions may be due to context effects and the awareness that depression symptoms are being assessed. Our primary objective was to evaluate differences in mean PHQ-8 somatic item sum scores (item 3 = sleep disturbances, item 4 = fatigue, item 5 = appetite changes) in people with SSc when the items were administered outside the context of a depression questionnaire versus as part of the PHQ-8. Our secondary objective was to evaluate differences in mean scores for each individual PHQ-8 somatic item when administered outside of or as part of the PHQ-8. We hypothesised that people who completed somatic items outside the context of a depression assessment would have significantly higher scores than those who completed the same items as part of a depression assessment.

## Methods

### Study design

We conducted a two-arm parallel superiority randomised controlled experiment with a 1:1 allocation ratio within the Scleroderma Patient-centred Intervention Network (SPIN) Cohort. The SPIN Cohort is a large multinational cohort that collects longitudinal data on patient-reported outcomes from people living with SSc (Scleroderma Patient-centered Intervention Network, 2024). Cohort participants complete a series of patient-reported outcome measures online every 3 months. In this experiment, when participants logged in to complete their routine assessment, they were randomly assigned to Reordered Items or Standard PHQ-8 arms. Participants in the Reordered Items arm completed a reordered version of the PHQ-8 in which the 3 PHQ-8 somatic items (item 3 = sleep disturbances, item 4 = fatigue and item 5 = appetite changes) were presented first, without any indication that they were part of a depression questionnaire, and the 5 psychological items were presented later in the assessment protocol.

The SPIN Cohort study was approved by the Research Ethics Committee of the Centre intégré universitaire de santé et de services sociaux du Centre-Ouest-de-l’Île-de-Montréal (#MP-05-2013-150) and by the ethics committees of all recruiting sites. Since the only change to routine cohort assessments was changing the order of item presentation, the Research Ethics Committee of the Centre intégré universitaire de santé et de services sociaux du Centre-Ouest-de-l’Île-de-Montréal determined that no additional ethics approval was needed and that SPIN Cohort participants did not need to be notified about the experiment.

We registered the experiment (ClinicalTrials.gov, NCT06772896) and posted a protocol on the Open Science Framework (https://osf.io/qkf8g/files/5kn49) prior to initiation. We reported results consistent with the Consolidated Standards of Reporting Trials statement (Moher *et al.*, 2010). Studies that use SPIN Cohort data have similar methods. Thus, the results from our experiment were reported as consistent with guidance from the Text Recycling Research Project (Hall *et al.*, 2021).

### Eligibility criteria

Eligible SPIN Cohort participants must be classified as having SSc based on 2013 American College of Rheumatology/European League Against Rheumatism criteria (van den Hoogen *et al.*, 2013) confirmed by a SPIN physician; aged ≥18 years; fluent in English, French, or Spanish; and have access to a computer or tablet with internet access. The SPIN Cohort is a convenience sample. Cohort participants are recruited during regular medical visits at SPIN recruitment sites (Scleroderma Patient-centered Intervention Network, 2024), and written informed consent is obtained. A medical form is submitted online by site personnel to enrol participants. Once online registration is completed, participants are sent an automated welcome email with instructions on how to activate their SPIN account and complete SPIN Cohort measures online. Cohort participants complete routine online assessments that last approximately 20 minutes upon enrolment and at 3-month intervals. All SPIN Cohort participants who logged in to the SPIN Cohort platform to complete their routine 3-month assessment during the period when the experiment was conducted were included.

### Experiment arms

Participants assigned to the Reordered Items arm received an assessment protocol in which the 3 PHQ-8 somatic items were presented separately from and prior to the 5 PHQ-8 psychological items without any indication that they were part of a depression symptom questionnaire. PHQ-8 psychological items were presented (1) later in the assessment and (2) with at least 1 other patient-reported outcome measure between the somatic items and psychological items. Apart from these 2 rules, as routinely done in SPIN Cohort assessments, all measures were presented in random order.

Participants assigned to the Standard PHQ-8 arm served as the control arm and completed the standard version of the PHQ-8, with the PHQ-8 and all other measures administered in random order. The title of the PHQ-8 was not presented to participants in either experiment arm; the PHQ-8 does not include instructions to respondents. PHQ-8 items were presented on 2 separate webpages in the Reordered Items arm and on a single webpage in the Standard PHQ-8 arm. The routine online assessment also contained 9 other measures, including the Health Literacy Survey Questionnaire (Finbråten *et al.*, 2018) and the Patient-Reported Outcomes Measurement Information System (Hays *et al.*, 2018), which included 8 measures.

### Randomisation and blinding

The SPIN Cohort platform was programmed to randomise each participant via simple randomisation in a 1:1 ratio to either the Reordered Items or Standard PHQ-8 arm. Randomisation occurred automatically and immediately when participants logged in to complete their routine assessment. Thus, study investigators were fully blind to participants’ assigned experiment arm. Since participants were not notified about the experiment, they were fully blind to the study objectives and their assigned experiment arm.

### Participant characteristics and experiment outcomes

SPIN physicians provided age, sex and medical information upon enrolment of participants in the SPIN Cohort. Participants reported sociodemographic data, including race or ethnicity, education level and marital status.

The primary outcome analysis compared the mean sum score of the 3 PHQ-8 somatic items (item 3 = sleep disturbances, item 4 = fatigue and item 5 = appetite changes) between participants in the two experimental arms. Secondary outcome variables were individual mean scores of the 3 PHQ-8 somatic items.

The PHQ-8 consists of 8 items that measure depression symptoms over the last 2 weeks. Items are scored on a 4-point scale ranging from 0 (not at all) to 3 (nearly every day), with higher scores (range 0–24) indicating more depression symptoms. The PHQ-8 has been shown to be equivalent to the PHQ-9 (Wu *et al.*, 2020), which is a valid measure of depression symptoms in SSc (Milette *et al.*, 2010). The PHQ-8 is available in English, French and Spanish (Arthurs *et al.*, 2012; Gómez-Gómez *et al.*, 2023). Only 2 studies have examined the minimal important difference of the PHQ-9 using anchor-based approaches, and they estimated a minimal important difference of between 2.0 and 3.7 points (Bauer-Staeb *et al.*, 2021; Kounali *et al.*, 2022). Many studies on the factor structure of the PHQ-9 have found that a one-factor model adequately explains item variance (Lamela *et al.*, 2020). However, other studies have suggested that a two-factor model, consisting of somatic and psychological latent factors, provides a better fit (Chilcot *et al.*, 2013). Somatic latent factors in two-factor models of the PHQ-9 include 3–5 items (Lamela *et al.*, 2020). The 3-item version consists of item 3 (sleep disturbance), item 4 (fatigue) and item 5 (appetite changes) (Chilcot *et al.*, 2013; Lamela *et al.*, 2020), which are commonly experienced in SSc. We performed a two-factor confirmatory factor analysis (CFA) to verify the 3-item somatic latent factor using SPIN Cohort data and found that the model fit well (Comparative Fit Index = 0.997; Tucker–Lewis Index = 0.995; Root Mean Square Error of Approximation = 0.058; Standardised Root Mean Square Residual = 0.036). See Appendix 1 for CFA methods and results.

### Statistical analysis

In August 2024, prior to initiating the experiment, we determined that 903, 910, 851 and 798 SPIN Cohort participants completed all measures in their scheduled assessments during the previous 4 3-month periods (August 2023–October 2023, November 2023–January 2024, February 2024–April 2024 and May 2024–July 2024). Assuming that at least 798 participants would log in to the SPIN Cohort platform to complete an assessment and be randomised, using a two-tailed test with a significance level of 0.05, we calculated that we would be able to detect an estimated effect size of 0.20 standardised mean difference, which is considered a small difference (Cohen, 1988), with 80% power.

Two-tailed between-groups *t*-tests with 95% confidence intervals (CIs) were used to compare the mean sum score of the 3 PHQ-8 somatic items (item 3 = sleep disturbances, item 4 = fatigue and item 5 = appetite changes) and mean scores of individual somatic items between participants in the Reordered Items and Standard PHQ-8 arms. We determined pre-experiment that analyses would be conducted as complete case analyses because we did not expect significant missing data. However, per our protocol, if >10% of randomised participants had not completed all PHQ-8 items, we would have conducted intent-to-treat analyses with missing data handled using multiple imputations by chained equations (Rubin, 1987; van Buuren and Groothuis-Oudshoorn, 2011). Analyses were conducted using the statistical software R (R Core Team, n.d.).

### Post hoc analysis

We conducted post hoc subgroup analyses at the request of a peer reviewer to explore the possible effects of sex (male and female), age (≤60 years and >60 years), SSc subtype (diffuse and limited) and language (English, French, Spanish) on our results. We used linear regression models to examine the interaction between each subgroup variable and our experiment arms.

## Results

The experiment was conducted during participant assessments from 17 January to 17 April 2025.

### Participants

**Figure 1.**
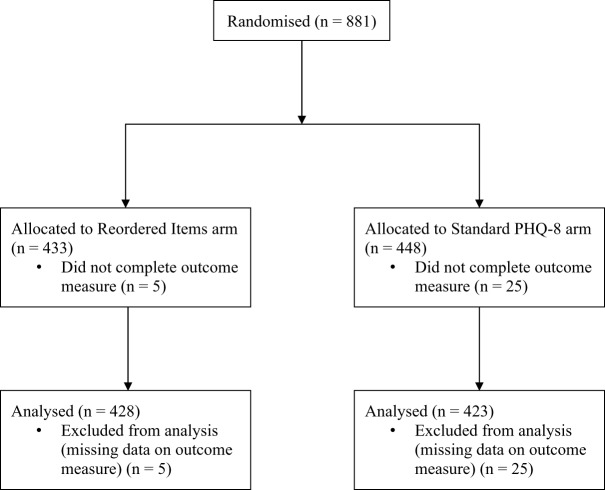
Participant flow diagram.

Of 881 SPIN Cohort participants who were randomised, 30 (3%) did not complete all PHQ-8 items and were excluded. Thus, 851 (97%) participants with complete PHQ-8 data were included in the analyses in the Reordered Items (*N* = 428) and Standard PHQ-8 (*N* = 423) arms. There were no substantive differences between participants who completed all PHQ-8 items and those who did not complete all items. See Appendix 2 for a comparison of characteristics between included and excluded participants. The flow of participants is presented in Figure 1.

Sociodemographic variables and disease characteristics are presented in Table 1. Mean (SD) age was 62.3 (11.8) years, 752 participants were female (88%), 703 participants identified as White (83%), 291 participants were classified as having diffuse SSc (34%) and mean (SD) time since onset of first non-Raynaud’s disease manifestation was 16.9 (9.8) years. Participants were from France (*N* = 326, 38%), the United States (*N* = 218, 26%), Canada (*N* = 197, 23%), the United Kingdom (*N* = 75, 9%) and Australia, Spain, or Mexico (*N* = 35, 4%). Characteristics of participants in the Reordered Items and Standard PHQ-8 arms were similar.

**Table 1.** Participant sociodemographic and disease characteristics at baseline

	Full sample		Reordered Items arm		Standard PHQ-8 arm	
	*N*^a^	Mean ± SD or N (%)	*N*	Mean ± SD or N (%)	*N*	Mean ± SD or N (%)
**Sociodemographic variables**						
Age, years	851	62.3 ± 11.8	428	62.5 ± 11.6	423	62.0 ± 12.0
Female sex	851	752 (88.4)	428	381 (89.0)	423	371 (87.7)
White race or ethnicity^b^	848	703 (82.9)	425	353 (83.1)	423	350 (82.7)
Education, years	844	15.6 ± 4.3	422	15.6 ± 4.7	422	15.6 ± 3.9
Marital status	844		423		421	
Married or living as married		579 (68.6)		283 (66.9)		296 (70.3)
Divorced or separated		111 (13.2)		55 (13.0)		56 (13.3)
Single		93 (11.0)		51 (12.1)		42 (10.0)
Widowed		61 (7.2)		34 (8.0)		27 (6.4)
Country	851		428		423	
France		326 (38.3)		160 (37.4)		166 (39.2)
United States		218 (25.6)		115 (26.9)		103 (24.3)
Canada		197 (23.1)		103 (24.1)		94 (22.2)
United Kingdom		75 (8.8)		29 (6.8)		46 (10.9)
Australia, Spain, Mexico		35 (4.1)		21 (4.9)		14 (3.3)
**Disease characteristics**						
Disease subtype	851		428		423	
Limited		528 (62.0)		265 (61.9)		263 (62.2)
Diffuse		291 (34.2)		145 (33.9)		146 (34.5)
Sine		32 (3.8)		18 (4.2)		14 (3.3)
Time since onset of first non-Raynaud’s disease manifestation, years	790	16.9 ± 9.8	399	17.5 ± 9.9	391	16.3 ± 9.6
Gastrointestinal involvement	844	724 (85.8)	426	373 (87.6)	418	351 (84.0)
Digital ulcers	816	97 (11.9)	411	52 (12.7)	405	45 (11.1)
Small joint contractures	809		407		402	
None or mild		607 (75.0)		310 (76.2)		297 (73.9)
Moderate		154 (19.0)		70 (17.2)		84 (20.9)
Severe		48 (5.9)		27 (6.6)		21 (5.2)
Large joint contractures	795		400		395	
None or mild		704 (88.6)		354 (88.5)		350 (88.6)
Moderate		74 (9.3)		37 (9.3)		37 (9.4)
Severe		17 (2.1)		9 (2.3)		8 (2.0)
History of renal crisis	841	24 (2.9)	424	11 (2.6)	417	13 (3.1)
Interstitial lung disease	749	232 (31.0)	377	108 (28.6)	372	124 (33.3)
Pulmonary arterial hypertension	819	55 (6.7)	413	33 (8.0)	406	22 (5.4)
Primary biliary cholangitis	825	15 (1.8)	416	7 (1.7)	409	8 (2.0)
**Overlap syndromes**						
Systemic lupus erythematosus	829	23 (2.8)	417	10 (2.4)	412	13 (3.2)
Rheumatoid arthritis	832	35 (4.2)	418	16 (3.8)	414	19 (4.6)
Sjögren’s disease	816	58 (7.1)	411	34 (8.3)	405	24 (5.9)
Autoimmune thyroid disease	815	49 (6.0)	411	24 (5.8)	404	25 (6.2)
Idiopathic inflammatory myositis	830	45 (5.4)	417	22 (5.3)	413	23 (5.6)

a Due to missing data, total sample size *N* < 851 for some characteristics.

b Race or ethnicity data were self-reported in each country using standard categories used in that country. Therefore, categories differed between countries.

### Outcomes

The mean (SD) PHQ-8 score was 6.0 (5.3) for the full sample, 6.0 (5.4) for Reordered Items arm participants and 6.0 (5.2) for Standard PHQ-8 arm participants. Outcome comparisons are presented in Table 2. The difference in PHQ-8 somatic item sum scores was not statistically significant (0.05 points; 95% CI −0.29 to 0.38). Differences in mean item scores were 0.04 points (95% CI −0.09 to 0.19) for item 3 (sleep disturbances), 0.03 points (95% CI −0.11 to 0.16) for item 4 (fatigue) and −0.03 points (95% CI −0.15 to 0.10) for item 5 (appetite changes).

**Table 2.** Differences in mean Patient Health Questionnaire-8 somatic item scores between the Reordered Items and Standard PHQ-8 groups

	Reordered Items arm	Standard PHQ-8 arm	
	Mean ± SD	Mean ± SD	Difference^a^ (95% CI)
**Primary outcome**			
Somatic items sum	3.25 ± 2.51	3.30 ± 2.41	0.05 (−0.29 to 0.38)
**Secondary outcomes**			
Item 3: Sleep disturbances	1.18 ± 1.04	1.22 ± 1.00	0.04 (−0.09 to 0.19)
Item 4: Fatigue	1.28 ± 0.98	1.31 ± 0.97	0.03 (−0.11 to 0.16)
Item 5: Appetite changes	0.79 ± 0.93	0.76 ± 0.93	−0.03 (−0.15 to 0.10)

CI, confidence interval.

a Differences between groups are calculated as Standard PHQ-8 – Reordered Items.

See Appendix 3 for post hoc analysis results. We did not find any significant subgroup interactions.

## Discussion

We examined whether administering PHQ-8 somatic items outside the context of a depression questionnaire would influence item scores among people with SSc, a chronic condition with substantial somatic symptom burden, including symptoms that overlap with PHQ-8 somatic items (sleep disturbance, fatigue, appetite changes). We did not find a statistically significant or substantive difference in mean PHQ-8 somatic item sum scores between participants who completed these items outside the context of a depression symptom assessment or as part of the standard PHQ-8. Our estimated difference of 0.05 points was 1.4%–2.5% of the minimal important difference (Bauer-Staeb *et al.*, 2021; Kounali *et al.*, 2022). There were no statistically significant or substantive differences in individual somatic item scores.

To the best of our knowledge, this is the first study to test the hypothesis that context effects may explain why people with chronic illnesses with substantial somatic symptom burden do not report more somatic items on self-report depression assessments like the PHQ-8 than people with similar psychological symptom scores but without a chronic illness. Our results suggest that findings from previous studies that did not find DIF in PHQ-9 scores (Leavens *et al.*, 2012; Jones *et al.*, 2015; Cook *et al.*, 2017; Hu and Ward, 2017; Marrie *et al.*, 2023) are not due to context effects.

Over 85% of SPIN Cohort participants have gastrointestinal symptoms. Previous studies have found that approximately 90% of people with SSc experience fatigue and that more than 75% have sleep difficulties (Bassel *et al.*, 2011; Willems *et al.*, 2014). It is possible that people with SSc may become accustomed to these symptoms over time, which would affect how they view their severity. In this case, they might view the same symptom presentation as less severe compared to someone without a chronic illness. Another possibility is that our findings reflect shared pathways in physical and mental disorders. Large bodies of evidence have linked depression to inflammation and immune symptom dysfunction, both of which are core elements to many chronic conditions (Pasco *et al.*, 2013; Miller and Raison, 2016; Beurel *et al.*, 2020; Troubat *et al.*, 2021), including SSc (Allanore *et al.*, 2015). It is possible that somatic symptoms of depression cannot be easily separated from similar somatic symptoms in SSc.

Our study has several strengths. We were able to include approximately 850 people, which provided sufficient power to detect even very small differences if they were present. Since we conducted our experiment as part of routine SPIN Cohort assessments, participants were not aware that an experiment was being done and were, thus, fully blinded to study conditions and our hypothesis. There are also limitations to consider. First, the SPIN Cohort is a convenience sample, and the outcome measure was completed online, which may reduce generalisability. However, participant characteristics from the SPIN Cohort have been shown to be comparable to those of other large SSc cohorts (Dougherty *et al.*, 2018). Furthermore, it is unlikely that differences in sociodemographic or SSc disease characteristics would alter the degree to which context effects might play a role in depression symptom assessment. Second, it is possible that some SPIN Cohort participants may have recognised PHQ-8 somatic items even when they were administered separately and without reference to the PHQ-8. We believe that this is unlikely since the PHQ-8 had not been administered as part of SPIN Cohort routine assessments since October 2020, over 4 years before our experiment. Furthermore, the individual PHQ-8 items are written in a way that does not give any indication that they refer to somatic symptoms related to depression or any other cause.

In summary, we conducted a randomised experiment to test whether context effects may explain why people with chronic illnesses do not appear to report more somatic symptoms on depression self-report measures compared to others without chronic illnesses with similar psychological symptom levels. Our results showed no difference in PHQ-8 somatic item scores when these were administered separately from cognitive and mood-related items versus as part of the standard PHQ-8. We did not find evidence that people’s knowledge that depression is being assessed influences how they report somatic symptoms of depression that may overlap with symptoms of physical illnesses.

## Supporting information

10.1017/S204579602610047X.sm001 Hu et al. supplementary material Hu et al. supplementary material

## Data Availability

De-identified participant data reported in this study will be made available upon request to the corresponding author.

## Supplementary material

The supplementary material for this article can be found at https://doi.org/10.1017/S204579602610047X.
