# Supplementary material for: Evaluating context effects on PHQ-8 somatic item scores among people with a chronic medical condition: a scleroderma patient-centred intervention network randomised experiment
Source: Epidemiol Psychiatr Sci. 2026 Feb 23;35:e11. doi: 10.1017/S204579602610047X (PMC12964067; doi:10.1017/S204579602610047X)
Supplement: Hu et al. supplementary material [file S204579602610047Xsup001.pdf]

APPENDICES

Appendix 1: Confirmatory factor analysis (CFA)

Methods

We performed a CFA on baseline PHQ-8 data from the SPIN Cohort (N = 2,252) to evaluate its two-factor structure with a 3-item somatic latent factor using R statistical software (R Core Team, n.d.). The 3 somatic items were item 3 (sleep disturbances), item 4 (fatigue), and item 5 (appetite changes). PHQ-8 item responses were modelled as ordinal Likert data using a weighted least squares estimator, a diagonal weight matrix, and robust standard errors. Model parameters were estimated using Nonlinear Minimisation with Box constraints. Model fit was assessed using a mean- and variance-adjusted chi-square test statistic, the Tucker-Lewis Index (TLI), the Comparative Fit Index (CFI), the Root Mean Square Error of Approximation (RMSEA), and the Standardised Root Mean Square Residual (SRMR). Since the chi-square test is highly dependent on sample size and can result in the rejection of a well-fitted model (Reise et al., 1993), we prioritised the TLI, CFI, RMSEA, and SRMR. A well-fitted model is generally characterised by a TLI and a CFI of ≥ 0.95, an RMSEA of ≤ 0.06, and an SRMR of ≤ 0.08 (Hu and Bentler, 2009). However, a TLI of ≥ 0.90 and an RMSEA of ≤ 0.08 are also commonly used as indicators of an acceptable model fit (Browne and Cudeck, 1992).

Results

Factor loadings on the Patient Health Questionnaire-8

Item	Factor loadings for somatic variables (95% CIs)	Factor loadings for psychological variables (95% CIs)
1. Little interest or pleasure in doing things	-	0.87 (0.86, 0.89)
2. Feeling down, depressed, or hopeless	-	0.89 (0.88, 0.91)
3. Trouble falling or staying asleep, or sleeping too much	0.74 (0.72, 0.77)	-

4. Feeling tired or having little energy	0.87 (0.85, 0.89)	-
5. Poor appetite or overeating	0.78 (0.76, 0.81)	-
6. Feeling bad about yourself - or that you are a failure or have let yourself or your family down	-	0.83 (0.81, 0.85)
7. Trouble concentrating on things, such as reading the newspaper or watching television	-	0.81 (0.79, 0.83)
8. Moving or speaking so slowly that other people could have noticed. Or the opposite - being so fidgety or restless that you have been moving around a lot more than usual	-	0.74 (0.71, 0.77)

CI = confidence interval.

Fit indices

CFI = 0.997; TLI = 0.995; RMSEA = 0.058; SRMR = 0.036.

References for Appendix 1

Browne MW and Cudeck R (1992) Alternative ways of assessing model fit. *Sociological Methods & Research* **21**(2), 230–258.

Hu L and Bentler PM (2009) Cutoff criteria for fit indexes in covariance structure analysis: conventional criteria versus new alternatives. *Structural Equation Modeling* **6**(1), 1–55.

R Core Team (n.d.) R: A language and environment for statistical computing. https://www.r-project.org/ (accessed 9 December 2024)

Reise SP, Widaman KF and Pugh RH (1993) Confirmatory factor analysis and item response theory: two approaches for exploring measurement invariance. *Psychological Bulletin* **114**(3), 552–566.

Appendix 2:

Baseline sociodemographic and disease characteristics for included and excluded participants

	Included participants		Excluded participants	
	N[a]	Mean ± SD or N (%)	N[b]	Mean ± SD or N (%)
**Sociodemographic variables**				
Age, years	851	62.3 ± 11.8	30	64.4 ± 10.8
Female sex	851	752 (88.4)	30	27 (90.0)
White Race or ethnicity[c]	848	703 (82.9)	30	24 (80.0)
Education, years	844	15.6 ± 4.3	29	15.9 ± 5.8
Marital status	844		30	
Married or living as married		579 (68.6)		25 (83.3)
Divorced or separated		111 (13.2)		3 (10.0)
Single		93 (11.0)		2 (6.7)
Widowed		61 (7.2)		0 (0.0)
Country	851		30	
France		326 (38.3)		14 (46.7)
United States		218 (25.6)		4 (13.3)
Canada		197 (23.1)		7 (23.3)
United Kingdom		75 (8.8)		2 (6.7)
Australia, Spain, Mexico		35 (4.1)		3 (10.0)
**Disease characteristics**				
Disease subtype	851		30	
Limited		528 (62.0)		17 (56.7)
Diffuse		291 (34.2)		13 (43.3)
Sine		32 (3.8)		0 (0.0)
Time since onset of first non-Raynaud's disease manifestation, years	790	16.9 ± 9.8	28	16.3 ± 10.5
Gastrointestinal involvement	844	724 (85.8)	30	27 (90.0)
Digital ulcers	816	97 (11.9)	29	2 (6.9)
Small joint contractures	809		28	

None or mild		607 (75.0)		18 (64.3)
Moderate		154 (19.0)		8 (28.6)
Severe		48 (5.9)		2 (7.1)
Large joint contractures	795		27	
None or mild		704 (88.6)		22 (81.5)
Moderate		74 (9.3)		4 (14.8)
Severe		17 (2.1)		1 (3.7)
History of renal crisis	841	24 (2.9)	29	0 (0.0)
Interstitial lung disease	749	232 (31.0)	27	7 (25.9)
Pulmonary arterial hypertension	819	55 (6.7)	28	0 (0.0)
Primary biliary cholangitis	825	15 (1.8)	29	0 (0.0)
**Overlap syndromes**				
Systemic lupus erythmatosus	829	23 (2.8)	29	0 (0.0)
Rheumatoid arthritis	832	35 (4.2)	29	0 (0.0)
Sjögren's disease	816	58 (7.1)	29	0 (0.0)
Autoimmune thyroid disease	815	49 (6.0)	29	1 (3.4)
Idiopathic inflammatory myositis	830	45 (5.4)	29	3 (10.3)

[a]Due to missing data, total sample size N < 851 for some characteristics. [b]Due to missing data, total sample size N < 30 for some characteristics. [c]Race or ethnicity data were self-reported in each country using standard categories used in that country. Therefore, categories differed between countries.

Appendix 3: Post hoc analysis results

	Linear regression coefficient estimates (95% CIs) per subgroup variable				
	Male sex: Reference = female	Age > 60 years: Reference = ≤ 60 years	Diffuse subtype: Reference = limited	French language: Reference = English	Spanish language: Reference = English
Reordered Item arm	0.02 (-0.33 to 0.37)	-0.15 (-0.66 to 0.36)	-0.11 (-0.52 to 0.30)	-0.13 (-0.59 to 0.32)	-0.13 (-0.59 to 0.32)
Subgroup variable	-0.67 (-1.38 to 0.04)	-0.43 (-0.91 to 0.04)	-0.15 (-0.64 to 0.35)	0.17 (-0.30 to 0.65)	-1.00 (-2.43 to 0.44)
Interaction Reordered Item arm * Subgroup variable	-0.64 (-1.67 to 0.38)	0.18 (-0.49 to 0.85)	0.19 (-0.51 to 0.89)	0.13 (-0.54 to 0.81)	1.33 (-0.85 to 3.51)

CI = confidence interval
